# Supplementary material for: Generative diffusion models for spatiotemporal influenza forecasting
Source: PLoS Comput Biol. 2026 Sep 30;22(9):e1014846. doi: 10.1371/journal.pcbi.1014846 (PMC13649142; doi:10.1371/journal.pcbi.1014846)
Supplement: Supplementary file 1 [file pcbi.1014846.s001.pdf]

Supporting Information for Generative diffusion models for spatiotemporal influenza forecasting

Joseph Lemaitre and Justin Lessler

1 Supporting Information

1.1 Evaluation of competing model formulations

To identify the factors that most influence Influpaint's forecasting performance, we conducted a series of one-at-a-time ablations around a reference configuration. The baseline used a 500-step cosine DDPM with a three-scale U-Net, the 30% surveillance / 70% simulated data mix, square-root scaling, no training-time enrichment, and the short-jump CoPaint schedule with no time travel. Forecasts were evaluated on 1–4-week horizons during the 2023–2024 and 2024–2025 FluSight seasons using the Weighted Interval Score (WIS).

Ablations varied diffusion depth and schedule, data composition, observation transforms, training enrichments, and inpainting schedules. We evaluated forecasting performance as the percent change in absolute WIS relative to the baseline (Supplementary Figure 1).

The number of diffusion steps had the largest effect: models trained with T=500 diffusion steps outperformed those trained with shorter (T=200) schedules. The choice of observation transform had the next-largest effect, with square-root scaling yielding better performance than linear scaling. Training-time enrichment (Poisson resampling, temporal padding, or intensity scaling) degraded performance, suggesting that such perturbations disrupt the inductive bias learned from the training data. In contrast, architectural variants within the same capacity class and alternative CoPaint schedules had minimal impact (Supplementary Figure 1).

Inpainting schedules evaluated. We evaluate three CoPaint/ODDIM sampling schedules that differ only in how strongly and how often the observed data are enforced along the reverse diffusion trajectory. *Time travel* means re-noising the current sample to a higher diffusion time and repeating the conditioned denoising process. When time travel is enabled, the *jump length* J defines the number of diffusion steps for each return. A *latent-refinement step* reduces the discrepancy between the predicted

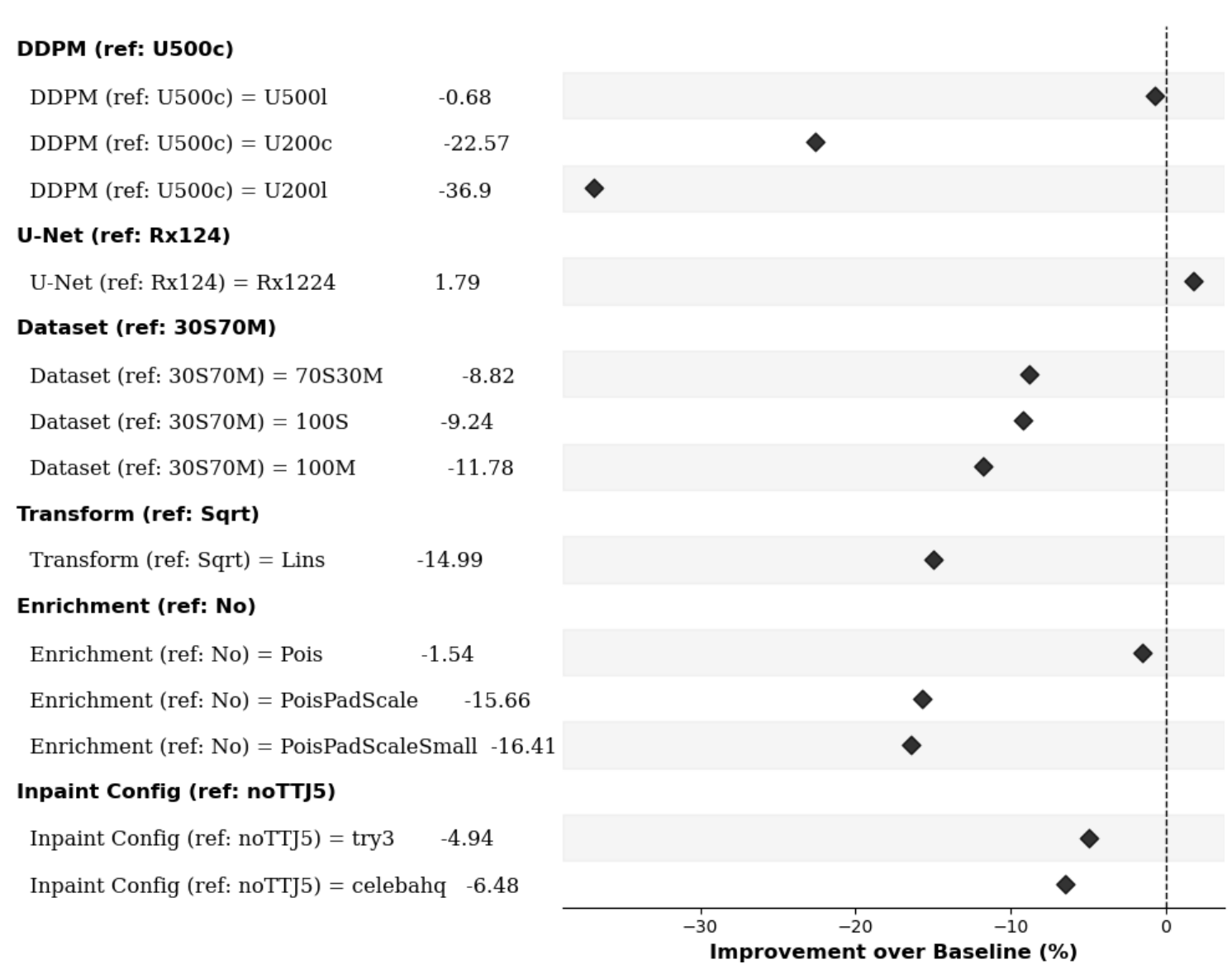

Supplementary Figure 1: Ablation effects on WIS. Each point shows the percentage improvement in summed absolute WIS for a model variant relative to the baseline configuration. Positive values indicate improved forecasting performance relative to the baseline, whereas negative values indicate worse performance. The first group reports diffusion-model denoising schedules, comparing 500 or 200 diffusion steps with either a linear (l) or cosine (c) variance schedule. The second group compares U-Net architectures, with *124* denoting a three-scale architecture with channel multipliers $(1, 2, 4)$ and *1224* a deeper four-scale architecture with channel multipliers $(1, 2, 2, 4)$. The dataset group compares training sets denoted $xxSyyM$, where $xx\%$ of samples come from surveillance data and $yy\%$ from modeled trajectories. The transform group compares square-root scaling of incidence values ($Sqrt$) with linear scaling ($Lins$). The enrichment group evaluates additional training perturbations: No (none), $Pois$ (Poisson resampling), $PoisPadScaleSmall$ (Poisson resampling with narrow temporal padding and intensity scaling), and $PoisPadScale$ (Poisson resampling with wide temporal padding and intensity scaling). The final group compares the three inpainting configurations described in the main text.

trajectory and the observed data. We compare the following schedules: (i) a short jump (J=5) with time travel and five latent-refinement steps per time index (`celebahq_try3`); (ii) the same jump length without time travel and with two latent-refinement steps (`celebahq_noTTJ5`); and (iii) a longer jump (J=10) with time travel and two latent-refinement steps (`celebahq`). Because conditioning is performed entirely at prediction time (the diffusion model is trained unconditionally), any surveillance/forecast mask can be used without retraining; this is consistent with theoretical results on resampling-based diffusion inpainting generalization [1]. The data used to evaluate predictive performance were not used for model training and are introduced to Influpaint only at generation time.

1.2 Relationship between training loss and forecasting performance

During training, Influpaint's loss (Supplementary Figure 2) measures performance on unconditional generation rather than directly measuring forecasting performance. When we compared this loss with forecasting skill, we found a positive correlation, though not a strict ordering across models (Supplementary Figure 3). This suggests that generative fit is informative about forecast quality, but does not fully determine it. Directly training diffusion models for forecasting may therefore improve performance further. Even so, these results show that training for unconditional generation already yields models with substantial forecasting skill.

1.3 Realized FluSight forecasts

We show a sample of real-time Influpaint forecasts submitted to the FluSight hub in Supplementary Figure 4.

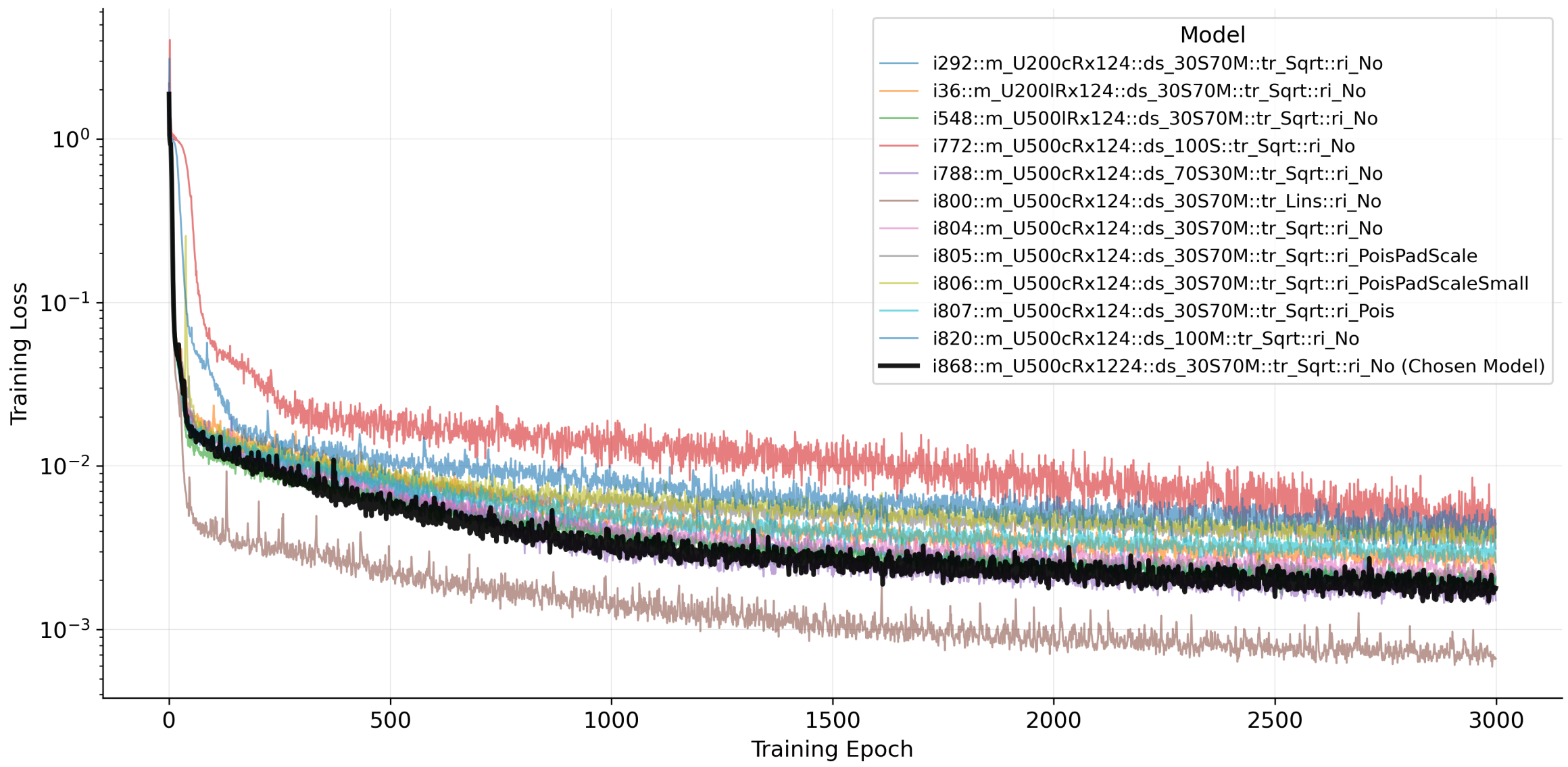

Supplementary Figure 2: Training losses during Influpaint calibration. Curves show the training loss over 3,000 epochs for the candidate model configurations evaluated during calibration. The model selected for downstream analyses, based on its combined ranking in relative and absolute WIS, is highlighted in black.

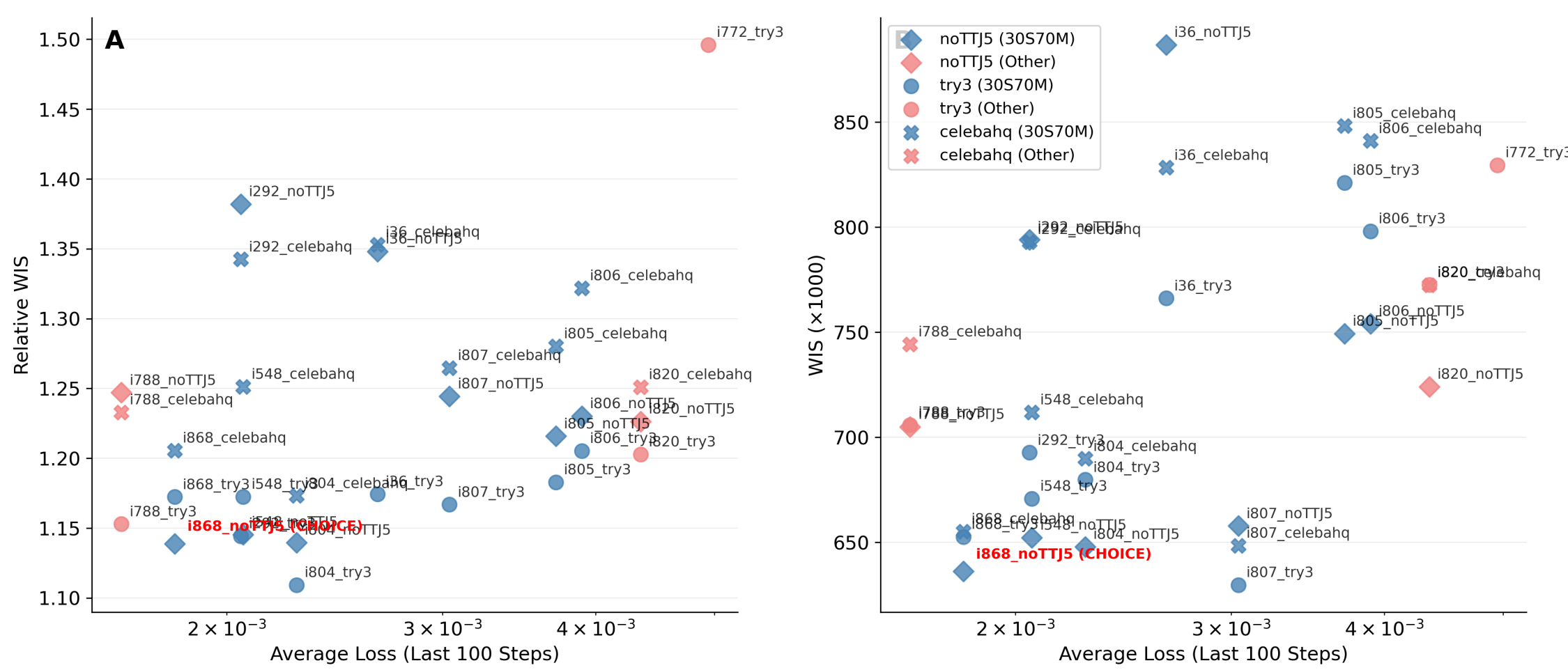

Supplementary Figure 3: Relationship between training loss and forecasting performance. Final training loss is compared with relative WIS (**a**) and absolute WIS (**b**) across candidate model configurations. Each point represents one model variant, and the configuration selected for downstream analyses is highlighted in red.

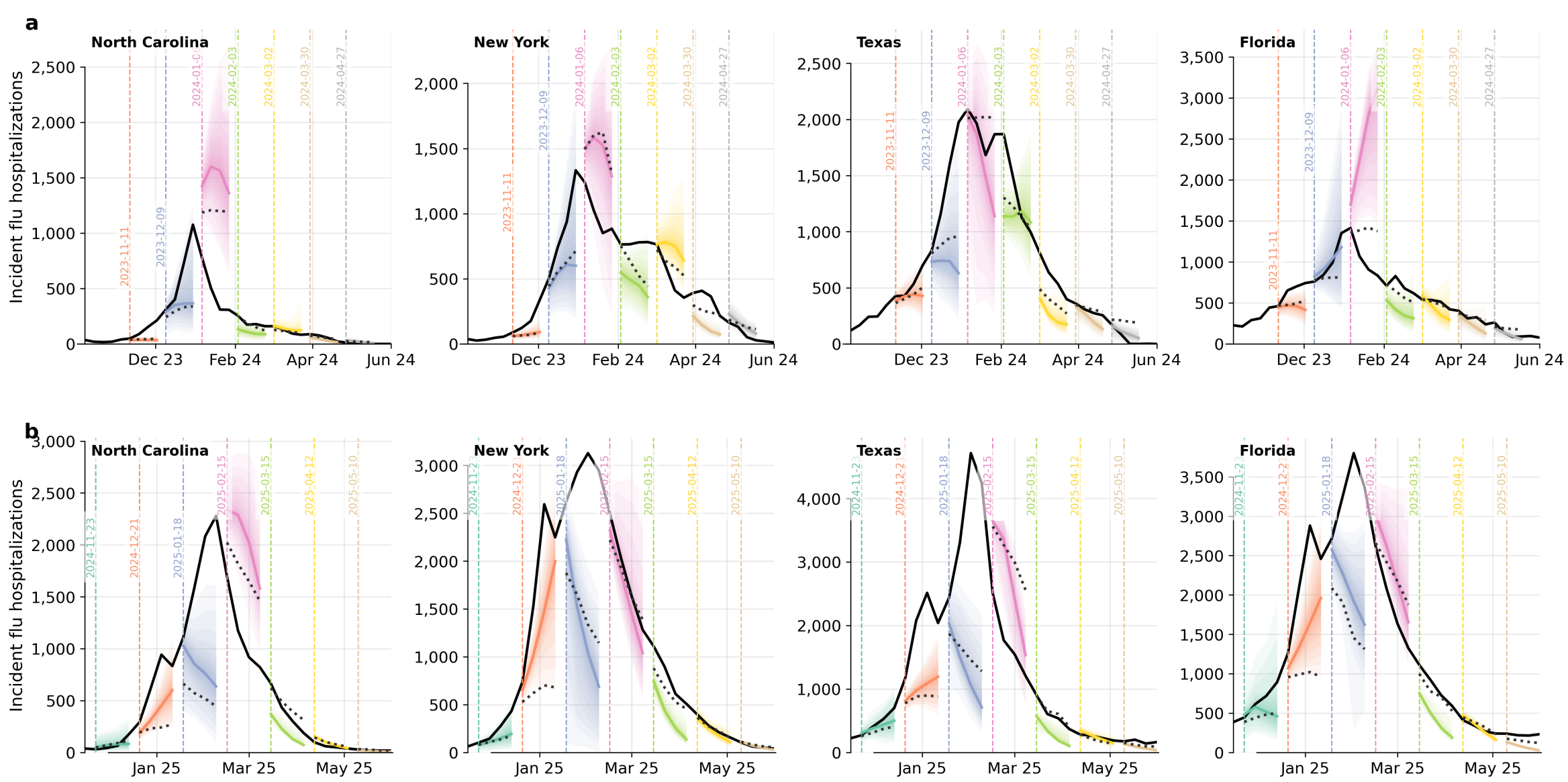

Supplementary Figure 4: a. Each panel shows submitted 4-week-ahead FluSight forecasts from `UNC_IDD-InfluPaint` for North Carolina, New York, Texas, and Florida at the same forecast dates used in **Figure 2** of the main text (colored dashed vertical lines). Forecast uncertainty is summarized by the submitted quantiles (colored fan) and median (colored line). The solid black line shows the observed final values. For the same forecast dates, the FluSight ensemble forecast is shown as a dotted line. **b.** Same as panel **a**, but for the 2024–2025 influenza season.

References

[1] Litu Rout et al. *A Theoretical Justification for Image Inpainting using Denoising Diffusion Probabilistic Models*. arXiv:2302.01217 [cs, math, stat]. Feb. 2023. DOI: 10.48550/arXiv.2302.01217. URL: http://arxiv.org/abs/2302.01217 (visited on 03/15/2023).
