# Supplementary material for: Generative diffusion models for spatiotemporal influenza forecasting
Source: PLoS Comput Biol. 2026 Sep 30;22(9):e1014846. doi: 10.1371/journal.pcbi.1014846 (PMC13649142; doi:10.1371/journal.pcbi.1014846)
Supplement: Supplementary file 2 [file pcbi.1014846.s002.pdf]

Response to Reviewers PCOMPBIOL-D-26-01592

Dear Dr. Lemaitre,

Two knowledgeable reviewers have provided detailed comments on your manuscript. Both are very positive overall, and I have therefore decided on minor revisions.

Please note that “minor” refers to the anticipated extent of the revisions, not to the care required in responding. Thoughtful expert reviewers are increasingly difficult to find, and I would not want to lose their engagement. I therefore ask that you consider all comments carefully and provide a clear, point-by-point response, including a brief explanation wherever you decide not to make a suggested change.

We are grateful to the editor and to anonymous reviewer #1 and Dr. Atamer Balkan for perpetuating the great academic tradition of peer-review and for the time and care put into these reviews. I am impressed by the review quality and the paper is significantly strengthened. Thank you. We respond point-by-point in the text below.

5) Thank you for stating "Influpaint submissions are available in the CDC repositories: https://github.com/cdcepi/Flusight-forecast-data for the 2022-2023 season and https://github.com/cdcepi/FluSight-forecast-hub for the 2023--2024 and 2024--2025 seasons, with model name UNC_IDD-InfluPaint." However, we were unable to access the sequence data using the information provided. Please review and update the Data Availability Statement to ensure that the underlying data are publicly accessible and that all accession numbers, links, or repository details are correct and functional.

I have updated the data availability statement and provide Influpaint submission in a simple zenodo archive, in addition to indicating the precise folder where the forecasts are stored. THe Data availability statement now states:
The pre-trained model weights used in this publication, along with all forecasts trajectories, model rankings, mode scores and mask experiment results are provided on Zenodo at 10.5281/zenodo.22699980. Influpaint real-time submissions are available in the CDC repositories https://github.com/cdcepi/Flusight-forecast-data for the 2022--2023 season (folder: data-forecasts and https://github.com/cdcepi/FluSight-forecast-hub for the 2023--2024 and 2024--2025 seasons (folder: model-output), with model name UNC_IDD-InfluPaint.

Reviewers' comments:

Reviewer #1:

Dear Editor and Authors,

In this manuscript, Lemaitre and Lessler demonstrate a influenza forecasting method based on diffusion models adapted from generative image modeling. An influenza season is represented by an image using time as one axis and location (US state) as the other. The color dimension represents influenza cases counts. This model is trained on both real flu timeseries and simulated data, and is then used to forecast trajectories using an "inpainting" algorithm. The model was submitted to the CDC FluSight challenge and performed better than the ensemble method.

I think this is a very interesting study and idea, and I only have a number of minor issues / questions / suggestions that should be addressed in the revised manuscript. I think the manuscript is easy to read and the code is extensively documented, making this a good starting point for future studies.

We thank the reviewer for this assessment and the careful reviews, and we are glad that the manuscript it easy to read as it was not easy to write.

I've listed some issues below. The most important issues I have are (1) that it is not clear if the model ranking is fair: Did influpaint get more recent data than the others? See my comment below (line 109). (2) It is not clear of the data used for evaluating the predictive performance also contains training data.
We answer below, but as a summary here: influpaint did not use more recent (future data) than another model, but present both real-time forecast and forecast from finalized ground-truth data. We have clarified the wording. Moreover, the data to evaluate predictive performance is not part of training data, we have clarified this as well.

Author Summary:

- Fix this sentence: "It is difficult to accurately forecasts of influenza transmission which makes it hard for public-health agencies to prepare and respond." -> It is difficult to accurately forecast influenza transmission...

Thank you, we have changed it to:

“*Accurately forecasting influenza activity is difficult, which complicates public-health preparedness and response*”

Introduction:

- line 49-50: "but are prone to create unrealistic emergent dynamics": It's not clear what you mean. Please explain this better.

Yes that is confusing, we have changed this to

Most forecasting approaches are based on mechanistic or statistical models. Mechanistic models incorporate epidemiological knowledge, but can generate unrealistic projections when the assumptions fail to describe an epidemic dynamic.

- line 53: "Both mechanistic and statistical approaches often under-represent the multimodal uncertainty seen in influenza seasons." It is not clear what you mean by multimodal uncertainty.
You most likely mean that the posterior predictive distribution has multiple modes. The reader might think that you are talking about uncertainty in a variety of measurements (or something else). Please clarify and add references.

Thank you, yes that was not clear, we have changed this sentence and added a citation

Frequently, forecast uncertainty involves distinct alternative futures (e.g a second peak or a decline) (Othus et al), and many mechanistic and statistical models under-represent the multimodality of these predictive distributions.

Results:

- Fig 1: Make 2 rows of panelsx and increase font size. Also, what do the line styles for the black curves mean. Add a legend.

Thank you, we have increased the font size and spread the figure over two rows, and added a legend for the past seasons. This comment has also made us realize that the font size was too small in most other figures, so we have increased it significantly.

- Fig 2: The "starting point" of the projections (colored fans) does not always match the observed data, and this is also true for the FluSight projections. Add a sentence to explain this.

Yes, you are correct, I think we used a quite unorthodox presentation so we have updated the figure to display how forecasts are usually presented:

- Left. old figure, the dashed line (e.g 2025-01-18 indicate the first line of the forecast, and we show how the forecast are without linking it to the last data point
- right : new figure, the dashed line is now the data cutoff for the forecast and we extend the forecast median line to go from this datapoint, which highlight what is the real trajectory forecasted. We have realized from your comment (and from R2 as well) that most papers use this representation.

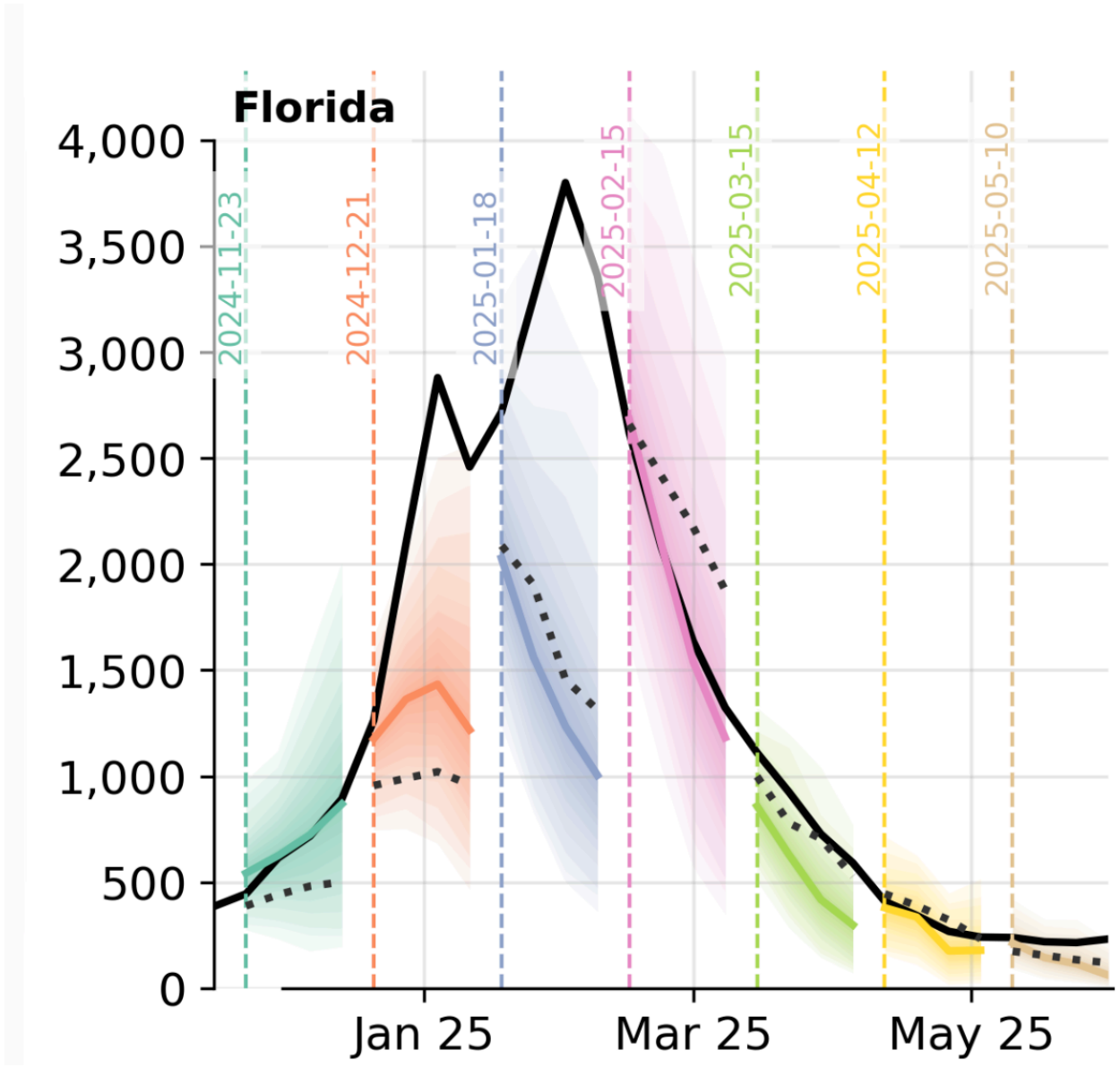

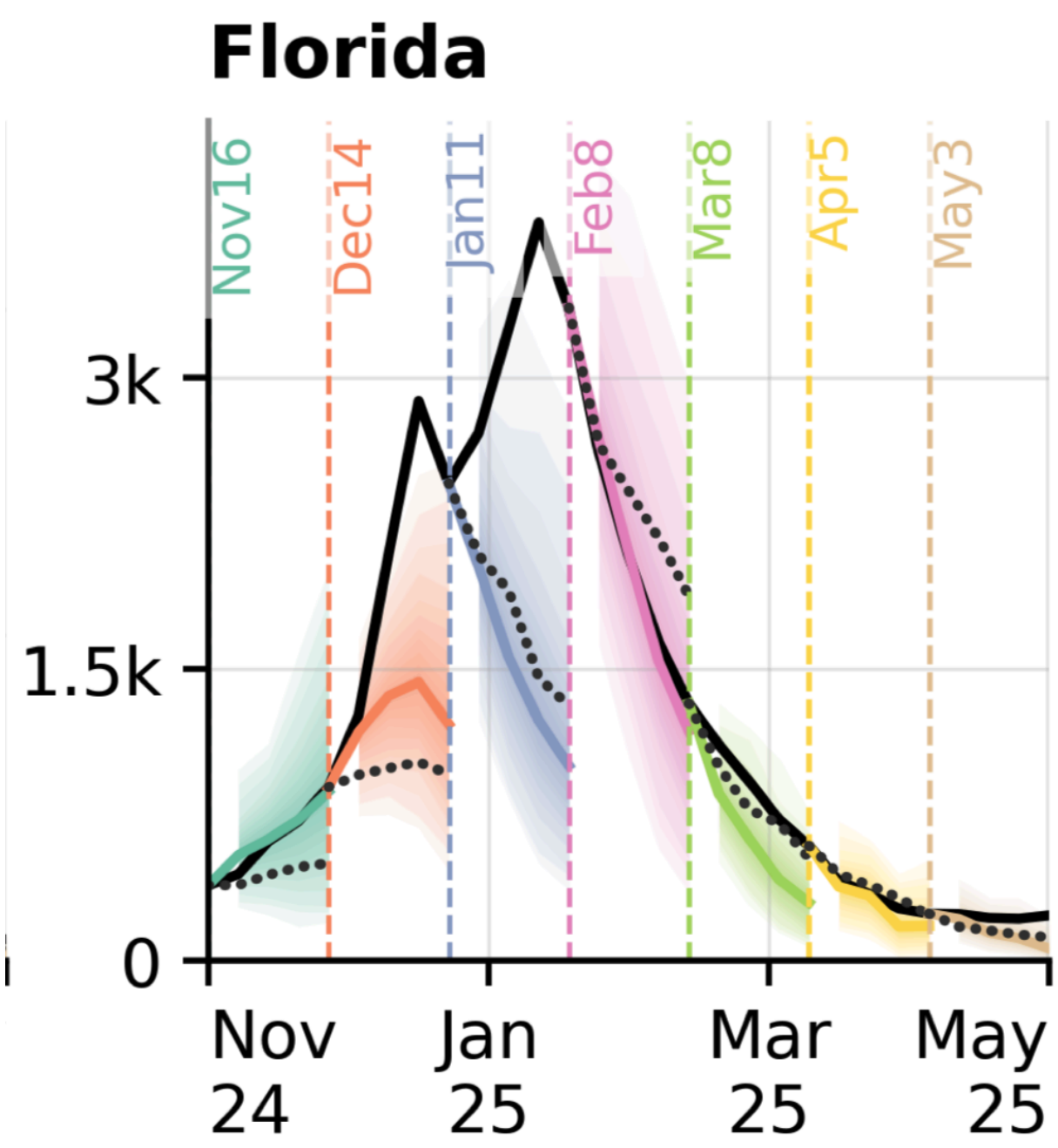

Under this representation, the discontinuity is not observed anymore, and we can more easily see why e.g the blue forecast for florida decreases. We have also updated the legend to state.

The dashed colored vertical lines marks the last week of observed data for a given forecast

- Paragraph line 99: This model is not the best model in the model ranking, but it is better than the ensemble model. This may sound a but odd for readers not familiar with FluSight model evaluations. Please say something about how this ensemble is generated. Is there anything to say about the better-performing models which might explain why they outperform influpaint?

We have added

The FluSight ensemble is an aggregation of submitted forecasts where each predictive quantile is the median of model submissions.

For better performing models, it is hard to explain it without opening a pandora box on model evaluation and the statistical power of single season evaluation. Not many models publishes their forecast year-over-year, and over a single reason the ranking may be very affected by that particular season features. The ensemble is the reliable and constant model across season, hence our comparison.

- Line 109: This is a bit concerning: Does this mean that the input data for forecasting is different between your model and the others? Or are you talking about a real-life application? Please explain. If you're using more (recent) data for you model predictions, then how do the rankings change if you take exactly the same datasets?

Thank you, this was confusing. We have clarified that we don't use future date for our projection but that this section is meant to evaluate influpaint forecasting performance on finalized data and directed the reader to the real-time comparison on the same data. The biggest change from this comment is that we added:

It is commonly regarded as among the best and most consistent available forecasting model. Because influpaint does not model reporting delay, this retrospective evaluation uses finalized, revised values for the observed weeks up to each forecast date. No observations from weeks after the forecast date are used to condition the forecasts. These inputs differ from the unrevised data available to

participating models in real time. \textbf{Section 2.4} reports Influpaint's operational performance using data available in real time.

- Line 116: The multi-modal quality of the predictions is not very well visualized by these plots. Could you add an inset with a histogram / kde of the predictions at a certain time point to clearly show this?
We added an inset in this plot showing how many model projection were increasing or decreasing 3 week after a given forecast, and we have added the corresponding descriptions and legend.

- Table 1: how can it be possible that the 30% modeling / 70% surveillance dataset has 1260 samples, while the 100% surveillance dataset has only 20 samples?
Good catch, we have clarified this by adding as the table legend:

$N_{samples}$ is the number of unique source trajectories before repetition. Both hybrid datasets contain 1,240 simulated and 20 surveillance samples. The mixing ratio is achieved by repeatedly sampling surveillance frames to obtain the desired proportion in the training dataset.

- Figure 4: Florida has a very high "baseline" incidence prior to the epidemic peak. Is this an outlier in your dataset, or is it more common? The model cannot capture this initial high incidence. Do you understand why?
Florida is an outlier, as we can see that e.g the other curves for this season starts much lower. We have added in the main text:

In Florida in 2023--2024, the early season incidence was exceptionally high compared to neighboring states, which Influpaint fails to capture under future-only conditioning (Figure~\ref{fig:masks}e).

Methods:
========

- Figure 5a: increase fontsize in inset.
Yes, we have increased the font size of the inset, along other font size to make this figure legible.

- Line 261: "The time and location axes have different semantics." I think this an important comment, and suggest that you address this earlier. Maybe discuss the possibility of alternative NN layers (as opposed to convolutions). I can imagine that some similarity structure between states exists that could be learned from the data itself. How do you determine the ordering of states on the y-axis? Is this based on e.g. spatial proximity? Or can (historical) surveillance data be used for an initial clustering of states? And does it matter?
Another paper where (genetic) outbreak data is converted into images and deep learning (CNNs, not diffusion) is this: https://doi.org/10.1371/journal.pcbi.1010598 and there the ordering of the sequence data is also quite important. Perhaps worth citing?
Very interesting paper indeed, it flew under my radar. In our case we have found no benefits to vary ordering, but we were wondering about other applications. We have and explanation and a citation:

Moreover, we found that the ordering of states along the location axis did not affect Influpaint's performance (Kupperman et al., using a different model, report that ordering influenced the performance of genetic HIV outbreak detection \cite{kupperman_deep_2022}). Influpaint's robustness may be explained by the flexibility of diffusion models, although we believe that incorporating inductive biases tailored to spatial epidemic dynamics could improve performance.

- Line 284: Define the acronym SMH
We have spelled out Flu Scenario Modeling Hub all three times instead of requiring the reader to memorize another acronym. Thank you.

- Line 320 (Eq 1). The data is sqrt-stransformed and scaled to the interval [0,2] (I'm assuming with a single, uniform scaling factor?) but how do you make sure that the noising and denoising step keep the data within this interval? Is the sampling truncated, or is there another unconstrained <-> constrained transform?

We have clarified the transform equation:

We applied square-root scaling before rescaling all frames to the $[0,2]$ range ($z = 2 \sqrt{x/M}$ where z is the transformed version of a dataset sample x, and M the dataset maximum).

And we have clarified that the intermediate and final steps are not constrained in [0,2]m, there is no clipping

Note that while the training data lie in the $[0,2]$ range, intermediate diffusion steps are not restricted to this interval.

- Line 353: "so the mask is always satisfied" This sounds a bit jargony to me. Please rewrite for a broader audience.

Yes, that even does not make any sens. We have clarified this by writing instead:

We first implemented RePaint \cite{lugmayr_repaint_2022_final}, a DDPM sampler that repeatedly overwrites the observed region during reverse diffusion (see Figure~\ref{fig:methods}\textbf{.C}). We design a mask indicating which part of the image is to be imposed (the past for a forecasting task) or generated (the future). As in the generative process above, each iteration starts from the current sample x_t, but before denoising one step backward, we replace the pixels with their ground-truth values noised to the same time step t, according to the mask.

- Line 350: The differences between RePaint and CoPaint are interesting, but I can't quite figure out what the differences are and why this improves the predictions from your text.

Is Figure 5C still an accurate cartoon of the CoPaint algorithm?

We have clarified that the figure 5C shows repaint and have highlighted the difference between CoPaint and RePaint

CoPaint \cite{zhang_towards_2023_final} addresses this [boundary] mismatch by introducing a Bayesian formulation that jointly updates both revealed and unrevealed pixels at every denoising step, ensuring global coherence and preserving the diffusion trajectory.

[...]

In our application, we find the transition between the observed (imposed) and generated part of a trajectory to be much smoother with CoPaint.

Unfortunately, the way the CoPaint algorithm addresses this boundary is very hard to distill without going in a lot of technical details.

SI:

===

Page 1 and 2: in the paragraph on "Inpainting schedules evaluated", it might help to first introduce definitions like "time travel" and "Jump length" and only then list the alternatives.

We have added the following definitions at the start of this paragraph:

\textit{Time travel}means re-noising the current sample to a higher diffusion times, to then repeats the conditioned denoising process. When time travel is enabled, the \textit{jump length} \(J\) defines the number of diffusion steps for each return. A \textit{latent-refinement step} reduces the discrepancy between the predicted trajectory and the observed data.

Page 2: "Because conditioning is performed entirely at prediction time (the diffusion model is trained unconditionally), any surveillance/forecast mask can be used without retraining" It's not clear if the data used for evaluating prediction was also used for model training. Please make sure you clarify this.

We have added the following clarification and clarified what we meant by this sentence:

Because conditioning is performed entirely at prediction time (the diffusion model is trained unconditionally), any surveillance/forecast mask can be used without retraining; this is consistent with theoretical results on resampling-based diffusion inpainting generalization \cite{rout_theoretical_2023}. The data used to evaluate predictive performance were not used for model training and are discovered by the Influpaint at the generation time.

General comments:
=================

- Overconfident predictions: Is this due to having trajectories without observation noise as input data? Would Poisson sampling of the prediction improve this? Your model probably predicts trajectories without observational noise.
We have found that adding poisson noise at training time to these trajectories degraded performance but increased the prediction interval (as WIS is divided into 3 components – over prediction, under prediction and spread – it penalize relatively heavily under confident prediction). We had not thought of adding noise to the generated trajectories and it seems a promising area, we will explore this for the coming season. Even though ideally we would like our diffusion process to capture confidence correctly by itself.

Thank you, anonymous reviewer #1, for your precious comments. We hope that we have answered to your question in a satisfactory manner.

Reviewer #2: To the authors of Generative diffusion models for spatiotemporal influenza forecasting

In this manuscript, the authors present the use of denoising diffusion probabilistic models for epidemic forecasting, describe their workflow and its forecasting performance, and present the performance of their model on a real-world forecasting challenge. The authors present a rigorous implementation from the computational science perspective, while at the same time writing the manuscript in a way that is accessible to both epidemiology and data science audiences. In that sense, I appreciate the work of the authors and enjoyed reading the manuscript. It is also very nice that the authors have presented their work in a relatively condensed manuscript. However, I think some parts would benefit from further explanation to make the work easier for the audience to follow:

Thank you, Dr. Atamer Balkan, for your assessment and we are glad you appreciated the paper format, it has been very hard to make this complex work interesting for several audience. Thank you for your thoughtful comments.

1. Between the Introduction and Results, I suggest including a short Methods Overview section, at least to guide readers through some of the terminology before they read and assess the results. I think the manuscript would benefit considerably from such a short section.
This is a great idea because we use a lot of concepts from the methods in the Results section before we introduced them, we have added this section that states:

Influpaint represents an influenza season as a matrix with weeks and locations as axes and incident influenza hospitalizations as values. It is trained on a collection of past seasons obtained from surveillance data and model simulations. Influpaint can then generate new seasons by progressively denoising random noise, which we call \textit{unconditional generation}. For forecasting, we use the same generation process but we impose observations from known weeks using a method called \textit{inpainting}, where a \textit{mask} indicates which entries are known. Further details are provided in \textbf{Materials and Methods}.

2. Figure 1 would benefit from some additional clarification for the reader. Could the authors please clarify the following points? (a) There are three black lines in each plot. Do they represent different seasons? If so, it would be helpful to label them accordingly. (b) I assume that the quantiles are shown by the colored areas in the background of each plot. If so, it would be useful to state this explicitly in the caption.
Thank you, point by point:
a) yes, we have added a legend, as mentioned as well by Reviewer #1
b) we have added in the caption for the quantiles:

The colored bands show the quantiles across 512 generated trajectories of weekly hospital admissions for California, New York, Texas, Florida, and Montana;

3. Line 88: The authors state that "Influpaint recovered much of the qualitative structure...". I think this conclusion may be slightly strong, considering that the mean inter-state correlation is almost half of the observed levels. I suggest reconsidering this statement.

First, while responding to your comment, I realized we had downplayed influpaint correlation from 0.455 to 0.646 (the actual observed being 0.833). The reason for this is that I was computing the correlation across the 64 locations in an image, but only the first 51 store actual data for US states, the rest is padding. This is still not enough to say *much* of the qualitative structure so we have changed this sentence to

*Likewise, Influpaint recovers **some** of the qualitative structure of multi-state epidemic timing …*

4. Line 103: What do the authors mean by "optimal conditions" here?

Yes, this is mean nothing, we have removed this sentence and added instead a more explicit sentence:

> *Although this comparison is biased because Influpaint forecasts were generated retrospectively using finalized values for observations up to each forecast date rather than under real-time reporting constraints,*

5. Lines 104–106: When the authors state, for example, "ranked 5th of 32", does 32 refer to the total number of different models included in the ensemble? If so, could the authors please add a brief note explaining this?

We added this sentence to reflect explain the ranking selection criteria

We compare InfluPaint against models that submitted to FluSight missing five or less forecasts: 32 models in 2023--2024 and 42 in 2024--2025.

We have to exclude model that misses too many forecast because as for WIS lower is better, missing a forecast produce a very good score for that week.

6. In Figure 2, the graphs show 4-week-ahead forecasts, but there is one aspect that I could not fully understand. Could the authors please explain the reason for the gaps between the last observed data points before the forecast period and the starting points of the forecast trajectories? One prominent example is Panel b for Florida, with forecasts starting on 2025-01-18. In forecasts, there is a substantial difference between the observed values immediately before the forecast period and the starting values of the forecast trajectories. Should we therefore understand these forecasts do not start immediately after the latest observed data point, but there is a reporting delay? Some additional explanation would be helpful here.

Yes, we had made a very confusing choice to show the forecast, I will redirect you to the response to reviewer 1 above, where I specifically screenshotted florida to explain that this gap is because the line were not placed on the last data point but on the first forecast point.

7. In Figure 3, in addition to the lines explained in the caption, there is another group of pinkish dotted lines spanning the full timeline. What do these lines represent?

These were the forecasted trajectory before their retrospective forecast date (despite the mask imposing past trajectories we can still see them under the main ground-truth curve). We have updated this plot to not show trajectories before the forecast dates as these are discarded anyway.

8. In Figure 4, Panel e: Is this past-only conditioning or future-only conditioning? Could the authors please edit the caption accordingly?

Good catch, it was future only conditioning, this was a typo. We have changed the caption in 4.e to

> *Reconstruction of the early season dynamics conditioned on later season observation for Florida.*

9. References: Almost half of the references in the reference list are preprints. Could the authors please check whether updated, published versions of these references are now available? For a few of the references I checked, I found that they had already been published in peer-reviewed journals or conference proceedings.

Indeed, we apologize for this oversight in this paper. Over 13 preprints, we have found 11 “peer-reviewed” versions and we have updated our bibliography accordingly. Thank you for spotting this.

10. I checked the GitHub repository (https://github.com/ACCIDDA/Influpaint) provided by the authors, but I was not able to reproduce the forecast results presented in the manuscript. I understand that training the model would not be feasible on a personal computer, but it would be very helpful if a user could regenerate forecasts for a small number of example states and seasons. Of course, my experience does not necessarily mean that the workflow provided on GitHub is not reproducible; however, as a user, I was not able to run the forecasting workflow successfully on my first attempt. The authors already note in the README file that the repository instructions should be updated to describe the use of CoPaint instead of RePaint. In addition to this update, I suggest providing easy-to-follow instructions that allow users to reproduce at least part of the analysis and generate forecast trajectories for a few example datasets. Having the repository updated during the publication process would be very helpful for both reviewers and future readers.

We are very sorry that the submitted version did not contain enough information to understand how to reproduce the paper. We have made a significant effort to improve this:

- First, a website https://accidda.github.io/influpaint/ with a walkthrough of the reproduction in 9 steps, each step is independent.
- We added the pre-trained model weights along with all forecasts trajectories, model rankings, mode scores and mask experiment results in a zenodo repository: https://zenodo.org/records/22699980.
- From this repository, with one command, you can regenerate all figures in the paper.
- Moreover, this repository provides the starting point of the 9 step in the walkthrough described above. So e.g if you want to skip the dataset creation step, you are able to do so by using directly the datasets provided in the zenodo. If you want to take directly the model weights to only do inpainting, you can do that as well.
- In each of the walkthrough steps a little box tells you how to reproduce, like this:

Paper reproducibility

You can bypass forecast generation for the selected model by getting its final outputs directly from the Zenodo reproducibility archive (archive README): the ensembles and forecast CSVs in `forecasts/retrospective/`, and the generated seasons in `forecasts/unconditional/`.

If you have not completed training or job preparation, you can start this stage with `i868::m_U500cRx1224::ds_30S70M::tr_Sqrt::ri_No::3000.pth` and `datasets/training/TS_30S70M_2025-07-17.nc` from the archive. Copy the NetCDF into `training_datasets/`, use scenario `868`, and pass the checkpoint path with `-m` in the command below instead of a training run ID. The matching training dataset is needed to reconstruct the model's transformations; the observation data described below are still required.

The saved retrospective forecasts cover 29 reference dates. They support selected-model analyses; rescoring the full candidate comparison also requires the other candidates' forecasts.

We have also provided this information in the paper, please let us know if you have encountered any problem. We would really love this to be reproducible.

Thank you, Dr. Atamer Balkan, for your thoughtful comments which I hope I have answered.
