# Supplementary material for: Generative diffusion models for spatiotemporal influenza forecasting
Source: PLoS Comput Biol. 2026 Sep 30;22(9):e1014846. doi: 10.1371/journal.pcbi.1014846 (PMC13649142; doi:10.1371/journal.pcbi.1014846)
Supplement: Peer review history

# Peer review: Decision Letter 0

**DOI:** 10.1371/journal.pcbi.1014846.r001

**Authors:** Thomas Leitner, Michiel van Boven

14 Aug 2026

PCOMPBIOL-D-26-01592

Generative diffusion models for spatiotemporal influenza forecasting

PLOS Computational Biology

Dear Dr. Lemaitre,

Two knowledgeable reviewers have provided detailed comments on your manuscript. Both are very positive overall, and I have therefore decided on minor revisions.

Please note that “minor” refers to the anticipated extent of the revisions, not to the care required in responding. Thoughtful expert reviewers are increasingly difficult to find, and I would not want to lose their engagement. I therefore ask that you consider all comments carefully and provide a clear, point-by-point response, including a brief explanation wherever you decide not to make a suggested change.

Please submit your revised manuscript by Sep 13 2026 11:59PM. If you will need more time than this to complete your revisions, please reply to this message or contact the journal office at ploscompbiol@plos.org. When you're ready to submit your revision, log on to https://www.editorialmanager.com/pcompbiol/ and select the 'Submissions Needing Revision' folder to locate your manuscript file.

Please include the following items when submitting your revised manuscript:

* A letter that responds to each point raised by the editor and reviewer(s). You should upload this letter as a separate file labeled 'Response to Reviewers'. This file does not need to include responses to formatting updates and technical items listed in the 'Journal Requirements' section below.

* A marked-up copy of your manuscript that highlights changes made to the original version. You should upload this as a separate file labeled 'Revised Manuscript with Track Changes'.

* An unmarked version of your revised paper without tracked changes. You should upload this as a separate file labeled 'Manuscript'.

If you would like to make changes to your financial disclosure, competing interests statement, or data availability statement, please make these updates within the submission form at the time of resubmission. Guidelines for resubmitting your figure files are available below the reviewer comments at the end of this letter.

As the corresponding author, your ORCID iD is verified in the submission system and will appear in the published article. PLOS supports the use of ORCID, and we encourage all coauthors to register for an ORCID iD and use it as well. Please encourage your coauthors to verify their ORCID iD within the submission system before final acceptance, as unverified ORCID iDs will not appear in the published article. Only the individual author can complete the verification step; PLOS staff cannot verify ORCID iDs on behalf of authors.

We look forward to receiving your revised manuscript.

Kind regards,

Michiel van Boven

Academic Editor

PLOS Computational Biology

Thomas Leitner

Section Editor

PLOS Computational Biology

**Journal Requirements:**

If the reviewer comments include a recommendation to cite specific previously published works, please review and evaluate these publications to determine whether they are relevant and should be cited. There is no requirement to cite these works unless the editor has indicated otherwise.

1) We ask that a manuscript source file is provided at Revision. Please upload your manuscript file as a .doc, .docx, .rtf or .tex. If you are providing a .tex file, please upload it under the item type u2018LaTeX Source Fileu2019 and leave your .pdf version as the item type u2018Manuscriptu2019.

2) Please upload all main figures as separate Figure files in .tif or .eps format. For more information about how to convert and format your figure files please see our guidelines:

https://journals.plos.org/ploscompbiol/s/figures

3) Please upload a copy of Figure Fig 1-5 which you refer to in your text on pages 3, 4, 5, 7, and 10. Or, if the figure is no longer to be included as part of the submission please remove all reference to it within the text.

4) Please send a completed 'Competing Interests' statement, including any COIs declared by your co-authors. If you have no competing interests to declare, please state "The authors have declared that no competing interests exist". Otherwise please declare all competing interests beginning with the statement "I have read the journal's policy and the authors of this manuscript have the following competing interests:"

5) Thank you for stating "Influpaint submissions are available in the CDC repositories: https://github.com/cdcepi/Flusight-forecast-data for the 2022-2023 season and https://github.com/cdcepi/FluSight-forecast-hub for the 2023--2024 and 2024--2025 seasons, with model name UNC_IDD-InfluPaint." However, we were unable to access the sequence data using the information provided. Please review and update the Data Availability Statement to ensure that the underlying data are publicly accessible and that all accession numbers, links, or repository details are correct and functional.

**Reviewers' comments:**

Reviewer's Responses to Questions

**Comments to the Authors:**

**Please note here if the review is uploaded as an attachment.**

Reviewer #1: Dear Editor and Authors,

In this manuscript, Lemaitre and Lessler demonstrate a influenza forecasting method based on diffusion models adapted from generative image modeling. An influenza season is represented by an image using time as one axis and location (US state) as the other. The color dimension represents influenza cases counts. This model is trained on both real flu timeseries and simulated data, and is then used to forecast trajectories using an "inpainting" algorithm. The model was submitted to the CDC FluSight challenge and performed better than the ensemble method.

I think this is a very interesting study and idea, and I only have a number of minor issues / questions / suggestions that should be addressed in the revised manuscript. I think the manuscript is easy to read and the code is extensively documented, making this a good starting point for future studies.

I've listed some issues below. The most important issues I have are (1) that it is not clear if the model ranking is fair: Did influpaint get more recent data than the others? See my comment below (line 109). (2) It is not clear of the data used for evaluating the predictive performance also contains training data.

Author Summary:

===============

- Fix this sentence: "It is difficult to accurately forecasts of influenza transmission which makes it hard for public-health agencies to prepare and respond." -> It is difficult to accurately forecast influenza transmission...

Introduction:

=============

- line 49-50: "but are prone to create unrealistic emergent dynamics": It's not clear what you mean. Please explain this better.

- line 53: "Both mechanistic and statistical approaches often under-represent the multimodal uncertainty seen in influenza seasons." It is not clear what you mean by multimodal uncertainty.

You most likely mean that the posterior predictive distribution has multiple modes. The reader might think that you are talking about uncertainty in a variety of measurements (or something else). Please clarify and add references.

Results:

========

- Fig 1: Make 2 rows of panels and increase font size. Also, what do the line styles for the black curves mean. Add a legend.

- Fig 2: The "starting point" of the projections (colored fans) does not always match the observed data, and this is also true for the FluSight projections. Add a sentence to explain this.

- Paragraph line 99: This model is not the best model in the model ranking, but it is better than the ensemble model. This may sound a but odd for readers not familiar with FluSight model evaluations. Please say something about how this ensemble is generated. Is there anything to say about the better-performing models which might explain why they outperform influpaint?

- Line 109: This is a bit concerning: Does this mean that the input data for forecasting is different between your model and the others? Or are you talking about a real-life application? Please explain. If you're using more (recent) data for you model predictions, then how do the rankings change if you take exactly the same datasets?

- Line 116: The multi-modal quality of the predictions is not very well visualized by these plots. Could you add an inset with a histogram / kde of the predictions at a certain time point to clearly show this?

- Table 1: how can it be possible that the 30% modeling / 70% surveillance dataset has 1260 samples, while the 100% surveillance dataset has only 20 samples?

- Figure 4: Florida has a very high "baseline" incidence prior to the epidemic peak. Is this an outlier in your dataset, or is it more common? The model cannot capture this initial high incidence. Do you understand why?

Methods:

========

- Figure 5a: increase fontsize in inset.

- Line 261: "The time and location axes have different semantics." I think this an important comment, and suggest that you address this earlier. Maybe discuss the possibility of alternative NN layers (as opposed to convolutions). I can imagine that some similarity structure between states exists that could be learned from the data itself. How do you determine the ordering of states on the y-axis? Is this based on e.g. spatial proximity? Or can (historical) surveillance data be used for an initial clustering of states? And does it matter?

Another paper where (genetic) outbreak data is converted into images and deep learning (CNNs, not diffusion) is this: https://doi.org/10.1371/journal.pcbi.1010598 and there the ordering of the sequence data is also quite important. Perhaps worth citing?

- Line 284: Define the acronym SMH

- Line 320 (Eq 1). The data is sqrt-stransformed and scaled to the interval [0,2] (I'm assuming with a single, uniform scaling factor?) but how do you make sure that the noising and denoising step keep the data within this interval? Is the sampling truncated, or is there another unconstrained <-> constrained transform?

- Line 353: "so the mask is always satisfied" This sounds a bit jargony to me. Please rewrite for a broader audience.

- Line 350: The differences between RePaint and CoPaint are interesting, but I can't quite figure out what the differences are and why this improves the predictions from your text.

Is Figure 5C still an accurate cartoon of the CoPaint algorithm?

SI:

===

Page 1 and 2: in the paragraph on "Inpainting schedules evaluated", it might help to first introduce definitions like "time travel" and "Jump length" and only then list the alternatives.

Page 2: "Because conditioning is performed entirely at prediction time

(the diffusion model is trained unconditionally), any surveillance/forecast mask can be

used without retraining" It's not clear if the data used for evaluating prediction was also used for model training. Please make sure you clarify this.

General comments:

=================

- Overconfident predictions: Is this due to having trajectories without observation noise as input data? Would Poisson sampling of the prediction improve this? Your model probably predicts trajectories without observational noise.

Reviewer #2: To the authors of Generative diffusion models for spatiotemporal influenza forecasting,

In this manuscript, the authors present the use of denoising diffusion probabilistic models for epidemic forecasting, describe their workflow and its forecasting performance, and present the performance of their model on a real-world forecasting challenge. The authors present a rigorous implementation from the computational science perspective, while at the same time writing the manuscript in a way that is accessible to both epidemiology and data science audiences. In that sense, I appreciate the work of the authors and enjoyed reading the manuscript. It is also very nice that the authors have presented their work in a relatively condensed manuscript. However, I think some parts would benefit from further explanation to make the work easier for the audience to follow:

1. Between the Introduction and Results, I suggest including a short Methods Overview section, at least to guide readers through some of the terminology before they read and assess the results. I think the manuscript would benefit considerably from such a short section.

2. Figure 1 would benefit from some additional clarification for the reader. Could the authors please clarify the following points? (a) There are three black lines in each plot. Do they represent different seasons? If so, it would be helpful to label them accordingly. (b) I assume that the quantiles are shown by the colored areas in the background of each plot. If so, it would be useful to state this explicitly in the caption.

3. Line 88: The authors state that "Influpaint recovered much of the qualitative structure...". I think this conclusion may be slightly strong, considering that the mean inter-state correlation is almost half of the observed levels. I suggest reconsidering this statement.

4. Line 103: What do the authors mean by "optimal conditions" here?

5. Lines 104–106: When the authors state, for example, "ranked 5th of 32", does 32 refer to the total number of different models included in the ensemble? If so, could the authors please add a brief note explaining this?

6. In Figure 2, the graphs show 4-week-ahead forecasts, but there is one aspect that I could not fully understand. Could the authors please explain the reason for the gaps between the last observed data points before the forecast period and the starting points of the forecast trajectories? One prominent example is Panel b for Florida, with forecasts starting on 2025-01-18. In forecasts, there is a substantial difference between the observed values immediately before the forecast period and the starting values of the forecast trajectories. Should we therefore understand these forecasts do not start immediately after the latest observed data point, but there is a reporting delay? Some additional explanation would be helpful here.

7. In Figure 3, in addition to the lines explained in the caption, there is another group of pinkish dotted lines spanning the full timeline. What do these lines represent?

8. In Figure 4, Panel e: Is this past-only conditioning or future-only conditioning? Could the authors please edit the caption accordingly?

9. References: Almost half of the references in the reference list are preprints. Could the authors please check whether updated, published versions of these references are now available? For a few of the references I checked, I found that they had already been published in peer-reviewed journals or conference proceedings.

10. I checked the GitHub repository (https://github.com/ACCIDDA/Influpaint) provided by the authors, but I was not able to reproduce the forecast results presented in the manuscript. I understand that training the model would not be feasible on a personal computer, but it would be very helpful if a user could regenerate forecasts for a small number of example states and seasons. Of course, my experience does not necessarily mean that the workflow provided on GitHub is not reproducible; however, as a user, I was not able to run the forecasting workflow successfully on my first attempt. The authors already note in the README file that the repository instructions should be updated to describe the use of CoPaint instead of RePaint. In addition to this update, I suggest providing easy-to-follow instructions that allow users to reproduce at least part of the analysis and generate forecast trajectories for a few example datasets. Having the repository updated during the publication process would be very helpful for both reviewers and future readers.

**********

**Have the authors made all data and (if applicable) computational code underlying the findings in their manuscript fully available?**

The PLOS Data policy requires authors to make all data and code underlying the findings described in their manuscript fully available without restriction, with rare exception (please refer to the Data Availability Statement in the manuscript PDF file). The data and code should be provided as part of the manuscript or its supporting information, or deposited to a public repository. For example, in addition to summary statistics, the data points behind means, medians and variance measures should be available. If there are restrictions on publicly sharing data or code —e.g. participant privacy or use of data from a third party—those must be specified.

Reviewer #1: Yes

Reviewer #2: Yes

**********

PLOS authors have the option to publish the peer review history of their article (what does this mean?). If published, this will include your full peer review and any attached files.

If you choose “no”, your identity will remain anonymous but your review may still be made public.

**Do you want your identity to be public for this peer review?**  For information about this choice, including consent withdrawal, please see our Privacy Policy.

Reviewer #1: No

Reviewer #2: **Yes:**  Büsra Atamer Balkan

[NOTE: If reviewer comments were submitted as an attachment file, they will be attached to this email and accessible via the submission site. Please log into your account, locate the manuscript record, and check for the action link "View Attachments". If this link does not appear, there are no attachment files.]

**Figure resubmission:**

**Reproducibility:**

To enhance the reproducibility of your results, we recommend that authors of applicable studies deposit laboratory protocols in protocols.io, where a protocol can be assigned its own identifier (DOI) such that it can be cited independently in the future. Additionally, PLOS ONE offers an option to publish peer-reviewed clinical study protocols. Read more information on sharing protocols at https://plos.org/protocols?utm_medium=editorial-email&utm_source=authorletters&utm_campaign=protocols

# Author comment: Author response to Decision Letter 1

**DOI:** 10.1371/journal.pcbi.1014846.r002

11 Sep 2026

Attachment Submitted filename: Response to Reviewers influpaint.pdf

# Editor report: Decision Letter 1

**DOI:** 10.1371/journal.pcbi.1014846.r003

**Authors:** Thomas Leitner, Michiel van Boven

17 Sep 2026

Dear Dr Lemaitre,

We are pleased to inform you that your manuscript 'Generative diffusion models for spatiotemporal influenza forecasting' has been provisionally accepted for publication in PLOS Computational Biology.

Before your manuscript can be formally accepted you will need to complete some formatting changes, which you will receive in a follow up email. A member of our team will be in touch with a set of requests.

Please note that your manuscript will not be scheduled for publication until you have made the required changes, so a swift response is appreciated.

IMPORTANT: The editorial review process is now complete. PLOS will only permit corrections to spelling, formatting or significant scientific errors from this point onwards. Requests for major changes, or any which affect the scientific understanding of your work, will cause delays to the publication date of your manuscript.

Should you, your institution's press office or the journal office choose to press release your paper, you will automatically be opted out of early publication. We ask that you notify us now if you or your institution is planning to press release the article. All press must be co-ordinated with PLOS.

Thank you again for supporting Open Access publishing; we are looking forward to publishing your work in PLOS Computational Biology.

Best regards,

Michiel van Boven

Academic Editor

PLOS Computational Biology

Thomas Leitner

Section Editor

PLOS Computational Biology

***********************************************************

Dear authors,

As both reviewers were already very positive about the initial submission, I have briefly checked your revisions and responses to their comments. I am satisfied that you have adequately addressed the points raised and do not consider another round of external review necessary.

I am therefore pleased to accept the manuscript for publication in PLOS Computational Biology as it stands.

Best wishes,

Michiel van Boven

# Editor report: Acceptance letter

**DOI:** 10.1371/journal.pcbi.1014846.r004

**Authors:** Thomas Leitner, Michiel van Boven

PCOMPBIOL-D-26-01592R1

Generative diffusion models for spatiotemporal influenza forecasting

Dear Dr Lemaitre,

I am pleased to inform you that your manuscript has been formally accepted for publication in PLOS Computational Biology. Your manuscript is now with our production department and you will be notified of the publication date in due course.

The corresponding author will soon be receiving a typeset proof for review, to ensure errors have not been introduced during production. Please review the PDF proof of your manuscript carefully, as this is the last chance to correct any errors. Please note that major changes, or those which affect the scientific understanding of the work, will likely cause delays to the publication date of your manuscript.

Soon after your final files are uploaded, unless you have opted out, the early version of your manuscript will be published online. The date of the early version will be your article's publication date. The final article will be published to the same URL, and all versions of the paper will be accessible to readers.

For Research, Software, and Methods articles, you will receive an invoice from PLOS for your publication fee after your manuscript has reached the completed accept phase. If you receive an email requesting payment before acceptance or for any other service, this may be a phishing scheme. Learn how to identify phishing emails and protect your accounts at https://explore.plos.org/phishing.

Thank you again for supporting PLOS Computational Biology and open-access publishing. We are looking forward to publishing your work!

With kind regards,

Sharmila Kamatchi

PLOS Computational Biology | Carlyle House, Carlyle Road, Cambridge CB4 3DN | United Kingdom ploscompbiol@plos.org | Phone +44 (0) 1223-442824 | ploscompbiol.org | @PLOSCompBiol
